# Quantitative Comparison of the Efficacy of Various Compounds in Lowering Intracellular Cholesterol Levels in Niemann-Pick Type C Fibroblasts

**DOI:** 10.1371/journal.pone.0048561

**Published:** 2012-10-29

**Authors:** Zachary T. Wehrmann, Tyler W. Hulett, Kara L. Huegel, Kevin T. Vaughan, Olaf Wiest, Paul Helquist, Holly Goodson

**Affiliations:** 1 Department of Chemistry and Biochemistry, University of Notre Dame, Notre Dame, Indiana, United States of America; 2 Department of Biological Sciences, University of Notre Dame, Notre Dame, Indiana, United States of America; International Centre for Genetic Engineering and Biotechnology, Italy

## Abstract

Niemann-Pick Type C disease (NPC) is a lethal, autosomal recessive disorder caused by mutations in the NPC1 and NPC2 cholesterol transport proteins. NPC’s hallmark symptoms include an accumulation of unesterified cholesterol and other lipids in the late endosomal and lysosomal cellular compartments, causing progressive neurodegeneration and death. Although the age of onset may vary in those affected, NPC most often manifests in juveniles, and is usually fatal before adolescence. In this study, we investigated the effects of various drugs, many of which modify the epigenetic control of NPC1/NPC2 gene expression, in lowering the otherwise harmful elevated intracellular cholesterol levels in NPC cells. Our studies utilized a previously described image analysis technique, which allowed us to make quantitative comparisons of the efficacy of these drugs in lowering cholesterol levels in a common NPC1 mutant model. Of the drugs analyzed, several that have been previously studied (vorinostat, panobinostat, and β-cyclodextrin) significantly lowered the relative amount of unesterified cellular cholesterol, consistent with earlier observations. In addition, a novel potential treatment, rapamycin, likewise alleviated the NPC phenotype. We also studied combinations of effective compounds with β-cyclodextrin; the addition of β-cyclodextrin significantly enhanced the cholesterol-lowering activity of vorinostat and panobinostat, but had mixed effects with rapamycin. Collectively, these results may provide a basis for the eventual development of improved NPC therapies.

## Introduction

Niemann-Pick Type C disease (NPC) is a lethal, autosomal recessive disorder resulting in abnormally high accumulations of cholesterol and other lipids in the late endosomes and lysosomes of many cell types [1]. Such symptoms are a result of faulty intracellular lipid trafficking involving the NPC1 and NPC2 proteins; in 95% of cases, the condition is due to mutations in the *NPC1* gene, while the other 5% are caused by mutated *NPC2* [2]. Symptoms in NPC-afflicted individuals are diverse, though they generally include hepatosplenomegaly, excessive sphingomyelin storage in parenchymal tissues, and some degree of progressive neurodegeneration. It is estimated that NPC occurs in approximately 1 in every 100,000 to 200,000 live births, and, though the age of onset and initial severity may vary among affected individuals, the disease is usually fatal before adolescence [1]. Unfortunately, an effective cure has yet to be discovered.

The mechanisms of cholesterol transport involving NPC1 and NPC2 are under current investigation, and much remains to be elucidated regarding their function. NPC1 has been identified as a membrane protein present in late endosomes and lysosomes, that, under normal circumstances, contributes to the removal of cholesterol from these compartments [3]. The NPC1 protein contains a transmembrane sterol-sensing domain, along with luminal domains specific for cholesterol binding and cholesterol-dependent NPC2 interaction, respectively [4]–[6]. NPC2, in contrast, has been identified as a glycosylated soluble endosomal and lysosomal cholesterol transporter that, through interactions with NPC1 and other proteins, also plays a vital role in managing intracellular cholesterol levels [1]. A model has been proposed in which NPC2 directly accepts cholesterol from LDL molecules in late endosomes and lysosomes, and delivers it to NPC1 for insertion into the membrane [5], [6]. Though the specific functions of these proteins and other factors involved in cholesterol management remain under investigation, improper functioning of either (or both) of these proteins is implicated in the development of NPC.

Though there now only exists one approved drug for the treatment of NPC–Miglustat, an inhibitor of glycosphingolipid synthesis certified for use in the European Union but not the United States–a variety of other drug treatments have been proposed to help fight the progression of NPC [7]. Currently, a major focus in the development of potential treatments is epigenetic modulators–compounds that regulate gene expression at the systemic and/or cellular levels. Theoretically, by increasing the expression of mutated genes that may retain some limited function, deleterious symptoms that otherwise result from decreased amounts of the fully functional protein could be diminished. With respect to NPC, this would mean increasing the amounts of mutated NPC1 or NPC2 protein with the hope that their suppressed functionality could be overcome by such an increase.

In support of this idea, the overexpression of mutated NPC1 has been shown to ameliorate the NPC phenotype in a cell line homoallelic for the mutation I1061T; this mutation represents roughly 15% of disease-causing alleles and is the most common mutation found in NPC patients from Western countries. [8], [9]. Additional relief has been demonstrated through increased expression of other factors responsible for cholesterol transport and removal processes, including but not limited to the Rab proteins. For example, Choudhury, *et al*. have demonstrated that overexpression of Rab7 and Rab9 in NPC cells significantly lowers intracellular cholesterol levels, while others have shown that increased levels of Rab4 and Rab8 produce similar results [10], [11].

Two classes of molecules that upregulate gene expression are histone deacetylase (HDAC) inhibitors and DNA methyl transferase (DNMT) inhibitors. The histone deacetylase family consists of enzymes involved in the modification of histones, proteins that regulate DNA condensation into chromosomes. In addition, HDAC’s act on a variety of other nuclear and cytoplasmic proteins [12]. HDAC inhibitors increase expression by preventing deacetylation of histone lysine residues, inhibiting DNA packing and thus enhancing transcription of many (but not all) genes [12]. DNMT inhibitors, in contrast, act to inhibit DNA methylation. When methylation is suppressed, expression of potentially silenced genes is increased [13]. Because both HDAC and DNMT inhibitors help increase gene expression, they are potential candidates for treating NPC; they may upregulate the expression of NPC1, NPC2, Rabs, or, more broadly, other factors that contribute to the management of intracellular cholesterol levels.

Previous work has indicated that the HDAC inhibitors vorinostat (suberoylanilide hydroxamic acid, SAHA; marketed as Zolinza for the treatment of cutaneous T cell lymphoma) and panobinostat (LBH-589; currently in Phase III trials for cutaneous T cell lymphoma) successfully alleviate the NPC phenotype in NPC mutant cells [14]. Vorinostat has also been shown to effectively cross the blood-brain barrier, a significant hurdle for treatments of the devastating neurological symptoms of NPC [15]. However, their therapeutic efficacy in combination with other drugs that display potential in treating NPC is not yet well understood. Likewise, the DNMT inhibitor decitabine (5-aza-2′-deoxycitidine; marketed as Dacogen for various myelodysplastic syndromes) has been shown to increase the expression of a variety of protein factors in myeloblastic leukemic cell lines with positive therapeutic results [16]. However, its effects on NPC cells have yet to be determined.

Potential treatments of NPC are not limited to these two specific classes of molecules, however. Several other drugs, many of which are already FDA-approved for other conditions, have been identified as agents that alter cholesterol homeostasis, and thus might have potential as NPC therapeutics. These FDA-approved compounds are especially attractive as potential therapeutic agents for NPC, as many drug development and approval phases can be bypassed, significantly expediting their introduction as NPC treatments.

β-cyclodextrin and its derivatives have been shown in previous studies to reduce intracellular cholesterol levels significantly in NPC1 and NPC2 mutants [17], and one derivative has been identified as a cholesterol-chelating agent that may mimic the NPC2 protein [18], [19]. It also has significant positive effects on a mouse model of NPC [20], and, in anecdotal instances, it has been shown to improve the neurological prognosis of NPC patients [21]. While the effects of β-cyclodextrin on NPC cells are well established, its cooperative effects with other therapies are not fully understood.

In addition, evidence has been presented that chloroquine and chlorpromazine may be potentially useful in treating NPC. Chloroquine has been implicated in inhibiting lysosomal proteolysis, which may help maintain NPC1 and NPC2 protein levels [8]. Chlorpromazine, meanwhile, has been identified as an agent that facilitates cellular cholesterol efflux to serum-based acceptors, and has been suggested as having value in the treatment of NPC [22]. However, both compounds have been shown to inhibit cholesterol esterification [23], [24], raising concerns over their potential to treat NPC.

Finally, rapamycin (sirolimus), an immunosuppressant, has been previously shown by Sharpe and Brown to decrease LDL-receptor gene expression, which may inhibit cholesterol uptake in treated cells [25]. It has also been demonstrated to stimulate cholesterol efflux in human mesangial cells previously induced by the cytokine IL-1β to retain cholesterol, suggesting a role in cellular lipid homeostasis [26]. Rapamycin has not yet been studied as a potential NPC therapy, but these observations suggest that it may have utility.

In light of these previous findings, we investigated the effect of each of these compounds–vorinostat, panobinostat, β-cyclodextrin, rapamycin, decitabine, chloroquine, and chlorpromazine–on intracellular cholesterol levels in NPC1 mutant fibroblasts. We determined their efficacy in lowering NPC cell cholesterol levels using a previously described procedure that quantifies cholesterol levels in lysosomes and late endosomes (referred to as lysosome storage organelles, or LSO’s) through fluorescence image analysis [27]. After identifying successful compounds, further studies were performed on the cholesterol-lowering effects of the successful drugs in combination with an effective concentration of β-cyclodextrin.

## Materials and Methods

The methods used in this study closely model those previously described by Pipalia, *et al*. to be useful in determining the cholesterol-lowering efficacy of various compounds in NPC mutant cells [27]. The process quantitatively determines intracellular cholesterol levels by means of fluorescent microscopy and imaging, and produces an “LSO Ratio” value indicating the amount of unesterified cholesterol relative to cholesterol levels in untreated solvent controls.

### Materials

Unless otherwise stated, all materials, including Eagle’s Minimal Essential Media (MEM), L-glutamine, fetal bovine serum (FBS), phosphate buffered saline (PBS), streptomycin, penicillin, DMSO, EtOH, filipin, paraformaldehyde (PFA), chloroquine, chlorpromazine, rapamycin, decitabine (5-aza-2′-deoxycitadine) and β-cyclodextrin were purchased from Sigma-Aldrich, Inc. (St. Louis, MO). Metamorph imaging software was from Molecular Devices (Downington, PA), ImageJ image processing and analysis software was from the National Institutes of Health (Bethesda, MD), and OriginPro statistical software was from Origin Lab Corp. (Northampton, MA).

### Cell Culture

Healthy GM00498 human fibroblasts (male, non-fetal skin cells aged 3 years) were acquired from the Coriell Institute for Medical Research (Camden, NJ). The mutant GM03123 human fibroblast cell line (aged 9 years, female) was also acquired from Coriell. GM03123 cells are compound heterozygotes that possess two point missense mutations– one CT at nucleotide 709, and another TC at nucleotide 3182 of the *NPC1* gene. These correspond to the substitutions P237S and I1061T (as mentioned, a very prevalent NPC1 mutation) in the luminal domains of the folded NPC1 protein, respectively [9]. Cells were cultured in 10% FBS/Eagle MEM solution, supplemented with L-glutamine, penicillin, and streptomycin, at 37°C and 5% CO_2_.

### Drug Solutions

The drugs studied–vorinostat (SAHA), panobinostat (LBH-589), decitabine, chloroquine, chlorpromazine, rapamycin, and β-cyclodextrin–were prepared in stock solutions of 20 mM, 300 µM, 20 mM, 200 mM, 80 mM, 240 µM, and 200 mM, respectively, and stored at −20°C. Vorinostat, decitabine, chlorpromazine, rapamycin, and β-cyclodextrin were dissolved in DMSO, while panobinostat was solubilized in EtOH, and chloroquine was dissolved in PBS. Stock solutions were diluted in media to the appropriate working solution concentrations.

### Cell Plating/Treatment Doses

Cells were treated in black-walled Costar 96-well polystyrene plates (Corning, Inc., Corning, NY). Prior to use, the plate wells were treated with 0.1% gelatin in PBS for 1 h at room temperature in order to increase cell adherence and thus prevent cells from detaching or washing off in later steps. Cells of interest were removed from culture conditions and diluted so that approximately 1750 cells would be present in each well, in order to achieve roughly 50–60% confluence upon imaging. 100 µL of cells were added to 100 µL of drug solution in media to reach a total well volume of 200 µL, with a range of concentrations for each tested compound. Proper solvent controls were run in parallel. The drugs that were identified as successful in the initial screen were tested in combination with 200 µM β-cyclodextrin, at all but the highest individually-tested concentration for each.

### Fixation and Staining

After incubation in drug treatments for the 48 h, cells were washed 2×200 µL with PBS and incubated for 45 minutes at 4°C in 3% PFA. Afterwards, the fixed cells were washed 1×200 µL with PBS, and treated with 50mM NH_4_Cl in PBS at room temperature to terminate fixation. They were then washed 2×200 µL with PBS, and treated with 100 µL 0.1 mg/mL filipin staining solution in PBS for 2 hours at room temperature. Filipin binds to unesterified cholesterol and fluoresces under UV excitation, allowing for identification of intracellular cholesterol inclusions. After staining, the cells were washed with 3×200 µL PBS prior to image acquisition.

### Fluorescent Microscopy

Images of each condition were acquired in 200 µL PBS using a Nikon TE2000 microscope (1X optivar, 10X/0.30NA bright field objective) and CCD Cascade 512B camera under UV excitation (340–380 nm, 40 nm dichronic, 430 nm long pass filter) using Metamorph (100 ms acquisition time, 1x binning) software. Twelve representative images of each condition (avoiding hyperconfluent clusters of cells) were acquired in each experiment.

### Image Processing and Analysis

A quantitative measure of intracellular cholesterol levels in drug-treated cells relative to untreated solvent controls was determined, and averaged across the 12 images of each condition, in the manner described by Pipalia, *et al.* [27]. ImageJ software was used to process and normalize images. After 5^th^ percentile background subtraction, contrasts on all images were adjusted to between 0 and 5000 units. Following these adjustments, low intensity thresholds–above which all cellular membranes are displayed–were set at 220 units/pixel, while the high thresholds–above which only cholesterol inclusions are visible–were set at 1200 units/pixel for all images. The total integrated pixel intensity above the high threshold was then divided by the total image area above the low threshold to yield the numerical LSO values. LSO ratio values were obtained by dividing the LSO value for a given treatment by the LSO value for that treatment’s solvent control. The resultant LSO ratio values represent the proportion of cholesterol levels in treated cells relative to levels in the controls. An LSO ratio of “1” represents cholesterol levels equal to those in the solvent controls, while an LSO ratio of “0” –signifying minimal unesterified cellular cholesterol–approximates levels in healthy fibroblasts.

### Statistical Analysis

The statistical significance of the cholesterol-lowering effects of the tested compound doses were determined using the one-way analysis of variance and Tukey’s Honest Significant Difference (HSD) post-hoc means comparison features of OriginPro software. Efficacies of the drugs tested in combination with β-cyclodextrin were analyzed using the two-way analysis of variance and Tukey’s HSD post-hoc means comparisons features of OriginPro.

## Results

**Figure 1:**
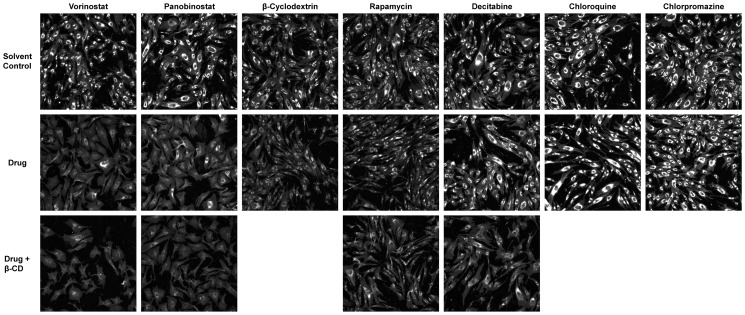
Representative Images of NPC1 Mutant Cells After Treatment with Various Compounds. GM03123 NPC1 mutant fibroblasts were treated with various drugs and combinations of drugs with 200 µM β-cyclodextrin for 48 hours, after which they were fixed, filipin stained, and imaged under UV excitation as described in Materials and Methods. Images are shown after contrast adjustment but prior to threshold application. The concentrations used in each displayed image for vorinostat, panobinostat, β-cyclodextrin, rapamycin, decitabine, chloroquine, and chlorpromazine were 10 µM, 75 nM, 200 µM, 7.5 nM, 0.625 µM, 50 µM, and 20 µM, respectively. Reduced cholesterol levels, as evidenced through decreased filipin fluorescence relative to solvent controls, are clearly observed in the vorinostat, panobinostat, β-cyclodextrin, and rapamycin treated cells. Further diminished fluorescence is observed in cells treated with both the drug and β-cyclodextrin (Drug + β-CD). No visibly obvious decrease in fluorescence is observed between cells treated with decitabine, chloroquine, or chlorpromazine, and their respective controls.

The methods used allowed us to determine the extent to which the tested drugs lowered intracellular unesterified cholesterol levels. During our initial screen of the drugs, in which their individual cholesterol-lowering efficacies were determined, several were found or reaffirmed to significantly lower intracellular cholesterol levels as demonstrated by their calculated LSO ratios. Representative images of NPC1 mutants exposed to treatments of each compound are presented in *Figure 1*.

### Effects of Vorinostat, Panobinostat, and Decitabine

**Figure 2:**
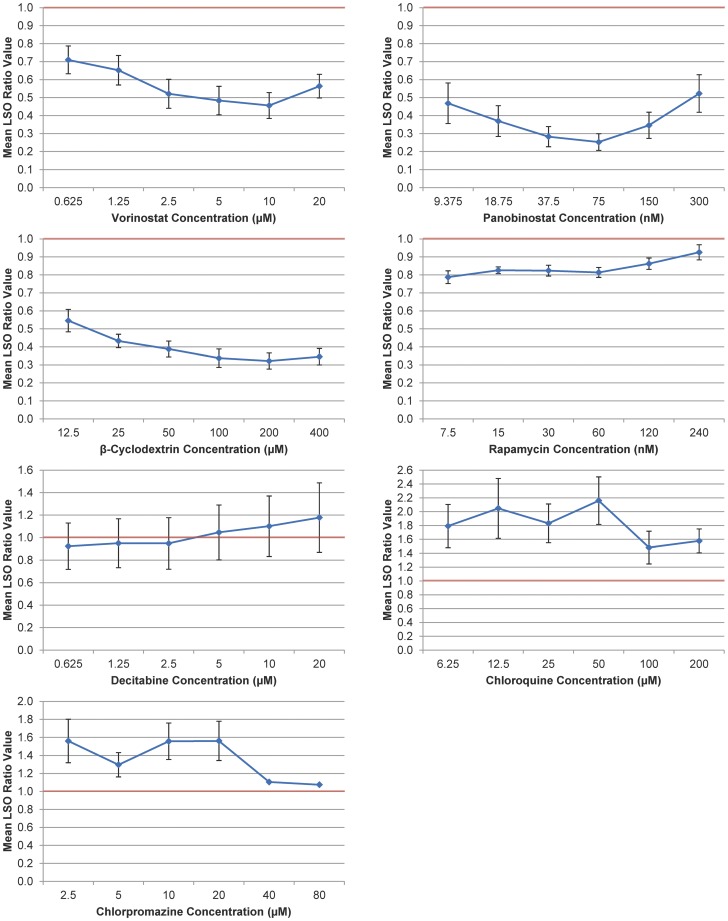
Dose Response Curves for Cholesterol-Lowering Efficacy as Demonstrated by LSO Ratio Values. NPC1 mutant fibroblasts were treated with various drugs for 48 h, after which they were fixed, filipin stained, imaged, and processed to give an LSO ratio value as described in Materials and Methods. Vorinostat, panobinostat, and β-cyclodextrin were very effective at lowering cholesterol levels, obtaining LSO ratios below 0.5 at several doses. Rapamycin was somewhat effective, consistently obtaining LSO ratios of ∼0.8. Decitabine had no significant effect on intracellular cholesterol levels. Chloroquine and chlorpromazine were not effective; they elevated cholesterol levels at all tested concentrations. The red lines indicate solvent controls (LSO ratio = 1). Each data point represents the average of at least three independent experiments totaling 36 images (three experiments×twelve images/experiment) and error bars are +/− SE. Data points without error bars reflect the results of one experiment; cells were not viable in two of three trials at chlorpromazine concentrations of 40 and 80 µM.

The compounds implicated in the modulation of gene expression displayed varying efficacies, with the two HDAC inhibitors–vorinostat and panobinostat–demonstrating the strongest effects (*Figure 2* and *Table 1*). Vorinostat, acting alone, was found to lower intracellular cholesterol levels to approximately 46% of those found in the parallel solvent controls at a concentration of 10 µM. Even at its least effective tested dose, 1.25 µM, vorinostat lowered levels by almost 35%. Panobinostat, meanwhile, lowered cholesterol levels by nearly 75% at a concentration of 75 nM, and, even at its least effective dose, 300 nM, lowered levels approximately 48%. It appeared that at concentrations greater than 75 nM, the cholesterol-lowering efficacy of panobinostat was diminished. The lone DNMT inhibitor tested, decitabine, displayed no significant effects at any concentration. Complete numerical and statistical data for these treatment conditions are presented in *Figure 2* and *Table 1*.

**Table 1:** Dose Dependence Summary Statistical Data.

Compound	Treatment Concentration	Mean LSO Ratio	Percent Cholesterol Reduction
Vorinostat	20 µM	0.563	43.7 **
	10 µM	0.456	54.5 ***
	5 µM	0.483	51.7 ***
	2.5 µM	0.521	47.9 ***
	1.25 µM	0.652	34.8 *
	0.625 µM	0.709	29.1
Panobinostat	300 nM	0.522	47.8 **
	150 nM	0.346	65.5 ***
	75 nM	0.252	74.8 ***
	37.5 nM	0.282	71.8 ***
	18.75 nM	0.369	63.1 ***
	9.375 nM	0.468	53.2 **
β-Cyclodextrin	400 µM	0.345	65.5 ***
	200 µM	0.321	67.9 ***
	100 µM	0.337	66.3 ***
	50 µM	0.388	61.2 ***
	25 µM	0.432	56.8 ***
	12.5 µM	0.545	45.5 ***
Rapamycin	240 nM	0.925	7.5
	120 nM	0.862	13.8 *
	60 nM	0.813	18.7 **
	30 nM	0.823	17.7 **
	15 nM	0.825	17.5 **
	7.5 nM	0.787	21.3 ***
Decitabine	20 µM	1.178	−17.8
	10 µM	1.101	−10.1
	5 µM	1.046	−4.6
	2.5 µM	0.948	5.2
	1.25 µM	0.950	5.0
	0.625 µM	0.923	7.7
Chloroquine	200 µM	1.577	−57.7
	100 µM	1.481	−48.1
	50 µM	2.158	−115.8
	25 µM	1.830	−83.0
	12.5 µM	2.047	−104.7
	6.25 µM	1.791	−79.1
Chlorpromazine	80 µM	1.074	−7.4
	40 µM	1.105	−10.5
	20 µM	1.561	−56.1
	10 µM	1.557	−55.7
	5 µM	1.296	−29.6
	2.5 µM	1.560	−56.0

* = significantly reduced from solvent control at p<0.05.

** = significant at p<0.01.

*** = significant at p<0.001.

### Effects of β-Cyclodextrin, Rapamycin, Chloroquine, and Chlorpromazine

The other tested compounds demonstrated varying degrees of effectiveness (*Figure 2* and *Table 1*). β-Cyclodextrin, at a concentration of 200 µM, was shown to lower intracellular cholesterol levels by approximately 68%, while, at a less effective dose of 12.5 µM, it still lowered levels by nearly 46%. Rapamycin also demonstrated success across several concentrations; at 7.5 nM, it lowered cholesterol levels by over 21%, while at 120 nM, it did so by approximately 14%. Curiously, the dose response curve for rapamycin was nearly flat, and the compound exhibited greater cholesterol reductions at the lower range of tested concentrations. Chloroquine and chlorpromazine were both unsuccessful at relieving the NPC phenotype, raising late endosomal and lysosomal cholesterol levels by amounts seemingly uncorrelated to the tested doses.

### Effects of 200 µM β-Cyclodextrin on Treatment with Vorinostat, Panobinostat, Rapamycin, and Decitabine

As stated earlier, due to their success in lowering NPC1 mutant cholesterol levels acting alone, vorinostat, panobinostat, and rapamycin were tested in combination with 200 µM β-cyclodextrin. For comparison purposes, the ineffective decitabine was also tested with β-cyclodextrin. Combinations of β-cyclodextrin and these compounds were significantly more effective than these compounds alone at lowering cholesterol levels (*Table 2*, Reduction from “Drug-Only”). More specifically, β-cyclodextrin enhanced the decreases in cholesterol observed through treatment with vorinostat alone by 26%–40%, and did so for panobinostat by 9%–22%. In addition, it increased the cholesterol-lowering efficacies of rapamycin and decitabine by 36%–40% and 35%–51%, respectively.

**Table 2:** Dose Dependence Summary Statistical Data for Treatments in Combination with 200 µM β-Cyclodextrin.

Compound	Treatment Concentration (+200 µM β-Cyclodextrin)	Mean LSO Ratio	Percent Cholesterol Reduction fromSolvent Control	Percent Cholesterol Reduction from 200 µM β-Cyclodextrin^1^	Percent Cholesterol Reduction from “Drug-Only” Result^2,3^
Vorinostat	10 µM	0.186	81.4 ***	13.5	27.0
	5 µM	0.223	77.7 ***	9.8	26.0
	2.5 µM	0.221	77.9 ***	10.0	30.0
	1.25 µM	0.264	73.6 ***	5.7	38.8
	0.625 µM	0.312	68.8 ***	0.9	39.7
Panobinostat	150 nM	0.256	74.4 ***	6.5	9.0
	75 nM	0.163	83.7 ***	15.8	8.9
	37.5 nM	0.179	82.1 ***	14.2	10.3
	18.75 nM	0.208	79.2 ***	11.3	16.1
	9.375 nM	0.246	75.4 ***	7.5	22.2
Rapamycin	120 nM	0.463	53.7 ***	−14.2	39.9
	60 nM	0.453	54.7 ***	−13.2	36.0
	30 nM	0.445	55.5 ***	−12.4	37.8
	15 nM	0.447	55.3 ***	−12.6	37.8
	7.5 nM	0.401	59.9 ***	−8.0	38.6
Decitabine	10 µM	0.587	41.3 ***	−26.6	51.4
	5 µM	0.599	40.1 ***	−27.8	44.7
	2.5 µM	0.603	39.7 ***	−28.2	34.5
	1.25 µM	0.574	42.6 ***	−25.3	37.6
	0.625 µM	0.546	45.4 ***	−22.5	37.7

*** = significantly reduced from solvent control at p<0.001.

1–LSO Ratio = 0.321; 67.9% cholesterol reduction.

2–See Table 1 for “Drug-Only” Results.

3–200 µM β-cyclodextrin significantly enhanced the cholesterol-lowering activity of all four compounds at p<0.001.

### Effects of Vorinostat and Panobinostat on Treatment with 200 µM β-Cyclodextrin

Combinations of vorinostat and panobinostat with β-cyclodextrin appeared to be slightly more effective at reducing cholesterol levels than β-cyclodextrin alone (which, as mentioned earlier, lowered cholesterol levels at 200 µM by 68%). A vorinostat/β-cyclodextrin treatment lowered intracellular cholesterol levels from control levels by 69%–81%, while a panobinostat/β-cyclodextrin combination did so by 74%–84% (*Table 2*). Vorinostat, thus, appeared to increase the efficacy of β-cyclodextrin by 1%–14%, and panobinostat did so by 7%–16% (*Table 2*, Reduction from 200 µM β-Cyclodextrin). However, while vorinostat and panobinostat appear to enhance cholesterol reductions by these small amounts when added to β-cyclodextrin, the differences that were observed between the cholesterol levels in these treatments and the β-cyclodextrin-only treatment are not statistically significant.

### Effects of Rapamycin and Decitabine on Treatment with 200 µM β-Cyclodextrin

**Figure 3:**
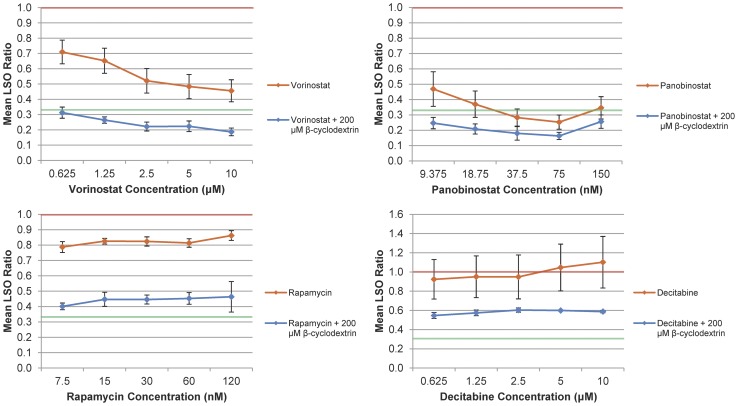
Dose Response Curves for Cholesterol-Lowering Efficacy of Tested Drugs in Combination with β-Cyclodextrin. NPC1 mutant fibroblasts were treated with vorinostat, panobinostat, rapamycin, and decitabine, both with and without 200 µM β-cyclodextrin, for 48 h, after which they were fixed, filipin stained, imaged, and processed to give an LSO ratio value as described in Materials and Methods. β-Cyclodextrin increased the impact vorinostat, panobinostat, rapamycin, and decitabine had on late endosomal and lysosomal cholesterol levels at all concentrations. The red lines indicate vehicle controls (LSO ratio = 1), while green lines indicate the average result of treatment with only 200 µM β-cyclodextrin (LSO ratio = 0.321). Each point represents the average of at least three independent experiments totaling 36 images (three experiments×twelve images/experiment) and error bars are +/− SE.

Curiously, treatments with β-cyclodextrin and either rapamycin or decitabine, respectively, were not as effective as treatment with solely β-cyclodextrin. Rapamycin/β-cyclodextrin combinations only lowered cholesterol levels by 54%–60%, while decitabine/β-cyclodextrin combinations did so by only 40%–45% (*Table 2*). Though rapamycin and decitabine each demonstrated a slight reduction or no effect on cholesterol levels acting alone, when used in combination with β-cyclodextrin, they served to elevate cholesterol levels by 8%–14% (rapamycin) and 23%–28% (decitabine) relative to those found with treatment with only 200 µM β-cyclodextrin (*Table 2*, Reduction from 200 µM β-Cyclodextrin). Rapamycin and decitabine thus appear to unexpectedly counteract β-cyclodextrin. Representative images from the combination treatments are also presented in *Figure 1*, and numerical data on all tested β-cyclodextrin combinations are displayed in *Figure 3* and *Table 2*.

## Discussion

In this report, we demonstrate the efficacy of several compounds and novel combinations thereof in treating NPC. Vorinostat and panobinostat, as expected, were confirmed to be agents that significantly lower intracellular cholesterol levels in NPC cells. Similarly, we confirmed β-cyclodextrin as a compound able to alleviate the NPC phenotype. More significantly, we observed that rapamycin, a drug that has not previously been tested in the context of NPC, lowered cholesterol levels in our tests. We also identified two drugs–chloroquine and chlorpromazine–that had previously shown some promise as treatment options in the scientific literature [8], [22], as unsuccessful cholesterol-lowering agents in this assay. It remains to be seen whether decitabine has any effect on NPC cells at a different range of concentrations, but in this study, it had no significant effect.

The fact that a novel drug, rapamycin, has now been shown to lower cholesterol levels opens up a new perspective from which to investigate NPC pathology and treatment. Rapamycin acts by inhibiting a well-conserved and extensively investigated serine-threonine kinase known as mTOR (molecular target of rapamycin). Inhibition of mTOR is achieved after rapamycin binds to another protein factor, FKBP12 [28]. mTOR plays multiple roles in cell physiology, regulating cell growth and cell proliferation, and responding to cellular nutrient and energy levels [28], [29].

There are multiple possible mechanisms by which rapamycin could affect cholesterol homeostasis in NPC cells. For instance, many mTOR downstream targets are involved with transcription and translation processes, such as the recruitment of ribosomes to mRNA [29]. As such, mTOR may play a role in modulating expression of proteins involved in cholesterol homeostasis. As noted above, rapamycin decreases LDL-receptor gene expression [25], which would be expected to reduce cholesterol uptake in treated cells. And, as mentioned, it stimulates cholesterol efflux in mesangial cells that have been induced to retain cholesterol [26]. Moreover, recent studies have shown that mTOR complex 1 (mTORC1) activates SREBP-1c (sterol regulatory element-binding protein 1c), which stimulates lipogenesis by upregulating the expression of genes associated with lipid synthesis [30], [31]. This observation suggests that rapamycin may inhibit intracellular cholesterol accumulation by reducing SREBP activity. The ability of rapamycin to reduce the intracellular cholesterol load, as seen in our assays, could result from any or all of these mechanisms, or alternatively, from the variety of other metabolic alterations induced by rapamycin. In any case, its observed cholesterol-lowering activity suggests that rapamycin might have therapeutic value.

Nonetheless, the dose dependence relationship observed for rapamycin treatments is somewhat unexpected. As is shown in *Figure 2* and *Figure 3*, the rapamycin dose dependence curve is nearly flat across the range of tested concentrations, and the cholesterol-lowering effects of rapamycin are actually more pronounced at lower dose levels (decrease of 21.3% at 7.5 nM), than they are at higher concentrations (insignificant decrease at 240 nM). If, however, rapamycin’s cholesterol-lowering effects are induced via the mTORC1/SREBP1c pathway, these observations are consistent with recent proposals that suggest mTORC1 is most strongly inhibited at low nanomolar (0.5–100 nM) concentrations [32]. It remains to be determined, though, whether or not the observed trend continues beyond our range of concentrations. These results indicate that further investigation of the impact of rapamycin on intracellular NPC cholesterol levels is warranted.

The second major finding of this study is that the addition of β-cyclodextrin to vorinostat, panobinostat, and rapamycin, significantly enhanced cholesterol reductions relative to the use of these three compounds individually. Though the specific manner of β-cyclodextrin’s cholesterol-lowering action remains to be fully elucidated–aside from its potential to mimic the NPC2 protein through a cholesterol-chelating mechanism [18], [19]–its observed effect may also be partly attributable to its ability to act as a drug carrier, facilitating the entry of vorinostat, panobinostat, and rapamycin into cells [33].

However, the β-cyclodextrin/rapamycin and β-cyclodextrin/decitabine combination therapies also had surprising aspects: these treatments resulted in higher cholesterol levels than those found from treatment with β-cyclodextrin alone. Given that rapamycin lowered cholesterol levels by approximately 20% at most concentrations when used alone, and that decitabine, individually, had no significant effect on cholesterol levels at any concentration, it would seem logical that their addition to a β-cyclodextrin treatment would be slightly beneficial or, at worst, have no impact. The observed results suggest that rapamycin and decitabine may in some way counteract the action of β-cyclodextrin, or somehow adversely affect lipid homeostasis in the presence of β-cyclodextrin. Given the intricacy of the mTOR pathway discussed above, it is possible that β-cyclodextrin interacts in some way with a downstream target of mTOR to affect cholesterol homeostasis. And, though decitabine’s mechanism of action–inhibition of DNA methylation–is not nearly as complicated as that of rapamycin and mTOR, it is also possible that there is some underlying opposing interaction between β-cyclodextrin and DNA methylation machinery. Our results indicate that future studies on these apparent counteractive effects are justified.

In summary, we have reaffirmed the efficacy of vorinostat, panobinostat, and β-cyclodextrin in reducing intracellular cholesterol levels in NPC fibroblasts, and have shown that a new compound, rapamycin, alleviates the NPC phenotype. While the effects of rapamycin were relatively small, this observation could have clinical relevance because of its established use as an immunosuppressant and its many well-studied cellular interactions. We have also identified chloroquine, chlorpromazine, and decitabine as unsuccessful cholesterol-lowering agents in this assay. Finally, we have demonstrated that β-cyclodextrin significantly increases the cholesterol-lowering effectiveness of various compounds *in vitro*. Given that many of these compounds are already FDA-approved for other conditions, we hope that these findings can help to justify and guide further research and trials, and contribute to the eventual discovery of an effective treatment for this devastating disease.
